# Scarless Removal of Large Resistance Island AbaR Results in Antibiotic Susceptibility and Increased Natural Transformability in Acinetobacter baumannii

**DOI:** 10.1128/AAC.00951-20

**Published:** 2020-09-21

**Authors:** Anne-Sophie Godeux, Elin Svedholm, Agnese Lupo, Marisa Haenni, Samuel Venner, Maria-Halima Laaberki, Xavier Charpentier

**Affiliations:** a CIRI, Centre International de Recherche en Infectiologie, Inserm, U1111, CNRS UMR5308, ENS de Lyon, Université Claude Bernard Lyon 1, Villeurbanne, France; b Université de Lyon, VetAgro Sup, Marcy-l'Étoile, France; c Unité Antibiorésistance et Virulence Bactériennes, Université de Lyon—ANSES Site de Lyon, Lyon, France; d Laboratoire de Biométrie et Biologie Evolutive, CNRS UMR5558, Université Claude Bernard Lyon 1, Villeurbanne, France

**Keywords:** AbaR, *Acinetobacter baumannii*, natural transformation, resistance island

## Abstract

With a great diversity in gene composition, including multiple putative antibiotic resistance genes, AbaR islands are potential contributors to multidrug resistance in Acinetobacter baumannii. However, the effective contribution of AbaR to antibiotic resistance and bacterial physiology remains elusive. To address this, we sought to accurately remove AbaR islands and restore the integrity of their insertion site. To this end, we devised a versatile scarless genome editing strategy. We performed this genetic modification in two recent A. baumannii clinical strains: the strain AB5075 and the nosocomial strain AYE, which carry AbaR11 and AbaR1 islands of 19.7 kbp and 86.2 kbp, respectively. Antibiotic susceptibilities were then compared between the parental strains and their AbaR-cured derivatives. As anticipated by the predicted function of the open reading frame (ORF) of this island, the antibiotic resistance profiles were identical between the wild type and the AbaR11-cured AB5075 strains. In contrast, AbaR1 carries 25 ORFs, with predicted resistance to several classes of antibiotics, and the AYE AbaR1-cured derivative showed restored susceptibility to multiple classes of antibiotics. Moreover, curing of AbaRs restored high levels of natural transformability. Indeed, most AbaR islands are inserted into the *comM* gene involved in natural transformation. Our data indicate that AbaR insertion effectively inactivates *comM* and that the restored *comM* is functional. Curing of AbaR consistently resulted in highly transformable and therefore easily genetically tractable strains. Emendation of AbaR provides insight into the functional consequences of AbaR acquisition.

## Abstract (precis)

With a great diversity in gene composition, including multiple putative antibiotic resistance genes, AbaR islands are potential contributors to multidrug resistance in Acinetobacter baumannii. However, the effective contribution of AbaR to antibiotic resistance and bacterial physiology remains elusive. To address this, we sought to accurately remove AbaR islands and restore the integrity of their insertion site. To this end, we devised a versatile scarless genome editing strategy.

## INTRODUCTION

Acinetobacter baumannii is responsible for health care-associated infections and concerns because of its intrinsic resistance to many antimicrobials and its ability to acquire resistance genes (1, 2). A potential contributor to multidrug resistance in A. baumannii is a genomic island, named AbaR, with a great diversity in gene content and carrying multiple putative antibiotic resistance genes (3, 4). The first report of AbaR described a large genomic island of 86 kbp in the epidemic A. baumannii strain AYE in 2006 (5). With a transposon backbone, AbaR islands are considered mobile genetic elements and present a modular structure (3, 4, 6). Although AbaR may contain up to 25 putative antibiotic resistance genes, most AbaR carry genes predicted to confer resistance mainly to aminoglycosides, tetracycline, and sulfonamide (4, 6, 7). Despite increasing genomic data, the actual contribution of AbaR-type islands to antibiotic resistance has not been fully evaluated. Deletion mutants of the AbaR27 island (originally published as Tn*AbaR23*) were previously obtained and revealed a restored susceptibility to sulfamethoxazole and a partial increased susceptibility to tetracycline (4, 8). The authors also suggested a paradoxical role of AbaR27 in increasing susceptibility to ciprofloxacin. However, deletions of AbaR27 were obtained by allelic replacement of this AbaR with the *aacC1* cassette, conferring resistance to gentamicin and confounded susceptibility testing against aminoglycosides. In addition, the original insertion site of AbaR was not restored, which prevented analysis of the physiological consequence of the insertion of AbaR in the targeted gene. AbaR islands are found inserted in the *comM* gene (4). This gene encodes a putative ATPase involved in natural transformation in a few transformable species, including nonpathogenic Acinetobacter baylyi (9, 10). Natural transformation allows bacteria to actively import exogenous DNA, and if the sequence of the imported DNA presents sufficient identity, it integrates into the recipient cell’s chromosome by homologous recombination (11, 12). Interestingly, the *comM* gene is not strictly essential for transformation, but its mutation decreases the transformation efficiency by 2 orders of magnitude (9, 10). A. baumannii and related species A. nosocomialis were described as transformable in 2013 (13, 14). In A. baumannii, natural transformation appears as a conserved trait among clinical and nonclinical isolates of human and animal origin (13, 15, 16), providing the bacteria with a major route for acquisition of antibiotic resistance genes.

In this study, we aim at assessing the contribution of AbaR islands to antibiotic resistance of two human clinical strains of the main global clone 1 bearing small and large AbaRs. We also analyzed the consequences of AbaR-mediated *comM* gene disruption on the transformation efficiency of these strains. We bring evidence that interruption of the *comM* gene by AbaR results in a decrease in natural transformation. Using genetic approaches, we further confirm a role for the *comM* gene in natural transformation in A. baumannii.

## RESULTS AND DISCUSSION

### Curing AbaR in A. baumannii using a scarless genome editing strategy.

**FIG 1:**
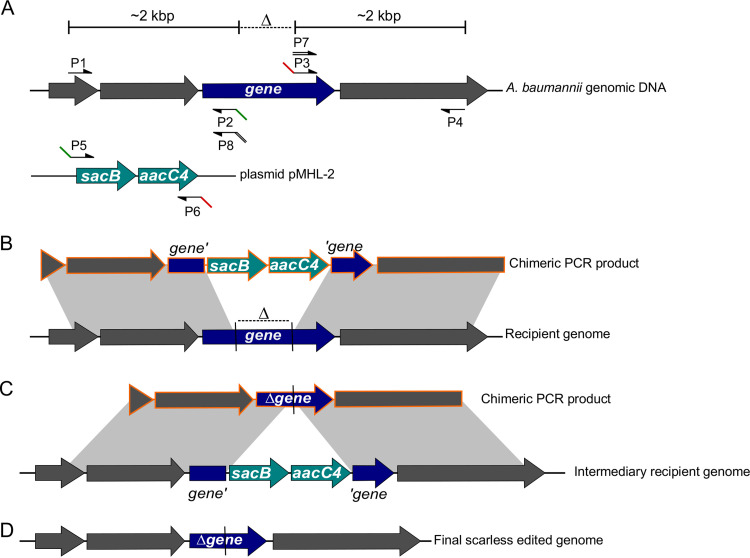
PCR-based genome editing of transformable A. baumannii. Schematic representations of steps for gene inactivation (dark blue gene) using natural transformation. (A) Annealing sites for primers of PCR assemblies on their respective templates: either A. baumannii genomic DNA for amplifications of 2 kbp upstream and downstream of the deleted region (Δ) or the laboratory plasmid pMHL-2 that bears the counterselectable cassette (light blue) with a *sacB* gene conferring sucrose sensitivity and the *aacC4* gene encoding resistance to apramycin. Note that primers P2, P3, and P7 share annealing sequences with primers P5, P6, and P8, respectively. (B) Chimeric PCR product (orange) obtained after two-step PCR assembly of products P1 + P2 with P5 + P6 and P3 + P4. This chimeric product bears the *sacB_aacC4* cassette flanked by two 2-kbp-long regions of homology with the recipient cell’s genome (gray areas). These homologous regions allow the recombination of the chimeric PCR product into the recipient cell’s genome, resulting, upon apramycin selection of the transformants, in the intermediary genotype represented in panel C. (C) Chimeric PCR product obtained after two-step assembly PCR of products P1 + P8 with P7 + P4 bearing the deleted gene flanked by homologous regions (gray areas), allowing recombination and replacement of the counterselectable cassette in the intermediary recipient genome. (D) The transformants are selected based on their sucrose resistance resulting in the final scarless edited genome.

**FIG 2:**
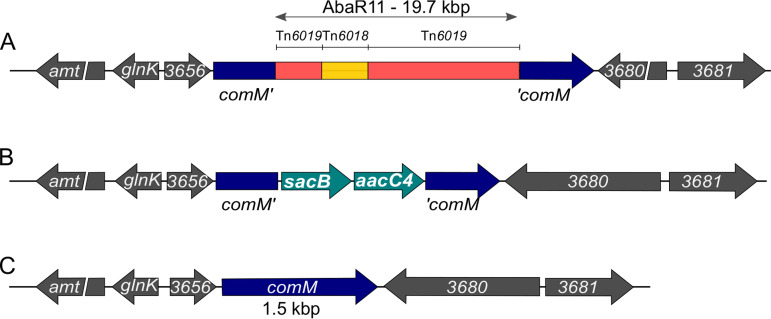
Genetic engineering of AbaR curing in A. baumannii. (A) Schematic representation of AbaR11 inserted in the *comM* gene in A. baumannii AB5075 genome. All the sequences and genes in gray color belong to the wild-type AB5075 chromosome, the interrupted *comM* gene is, however, highlighted in dark blue. Transposons Tn*6019* and Tn*6018* constituting AbaR11 are indicated as in reference 4. For illustration purposes, the scale of the AbaR island is not respected. (B) Intermediary genetic construct with the counterselectable cassette replacing the AbaR11 island. (C) Final genetic structure of the locus after restoring the *comM* gene integrity.

To cure A. baumannii of the AbaR island while restoring the original insertion site, we devised a scarless genome editing strategy. This method, depicted in Fig. 1, allows deletion, insertion, or single-base mutation. In contrast to other methods, it does not require cloning or prior genetic engineering of the target strain (17–19). Rather, it relies on the use of chimeric PCR products and takes advantage of natural transformation, a phenotypic trait exhibited by most isolates of A. baumannii. The method is based on a two-step selection (Fig. 1): first the introduction of a selectable/counterselectable *sacB_aacC4* cassette at the loci of interest (Fig. 1B), and then the replacement of the cassette by the desired sequence (Fig. 1C). First, a chimeric PCR product is assembled, consisting of the *sacB_aacC4* cassette bearing a selection marker (*aacC4*) conferring resistance to apramycin, flanked by 2-kbp-long sequences identical to the target region (Fig. 1B). This chimeric PCR product is introduced by natural transformation, and A. baumannii transformants are selected using apramycin, an antibiotic to which most clinical isolates are sensitive. A second PCR product is assembled, still carrying the homologous flanking regions but whose sequence is designed to produce deletion, insertion, or single-base mutation (deletion in Fig. 1C). Genetic replacement and the loss of the previously inserted *sacB* gene is then selected using sucrose resistance selection. We applied this method to delete AbaR11 in strain AB5075. The strain AB5075 is an A. baumannii ST1 strain isolated from a bone infection in 2008 with multidrug resistance (aminoglycosides, fluoroquinolones, and β-lactams, including carbapenems) (20). This strain bears a 19.7-kbp-long AbaR11 inserted in the *comM* gene with 19 predicted genes corresponding to five modules found in other AbaRs (Fig. 2A) (4, 8, 21). In a previous study, we identified the strain AB5075 as naturally transformable (15). Using natural transformation of PCR chimeric products, the AbaR island was replaced upon genetic recombination by the *sacB_aacC4* cassette (Fig. 2B). Then, the strain bearing the *sacB_aacC4* cassette in the *comM* gene was subjected to transformation with an assembly PCR encompassing the insertion region cured of AbaR, thereby carrying an intact *comM* gene. Then we performed genetic analysis of sucrose-resistant and apramycin-susceptible clones to select an AbaR-cured strain (named AB5075-R) with a restored *comM* gene presumably encoding a full-length ComM protein (Fig. 2C).

### Resistance profiles associated with AbaR curing in two clinically relevant multidrug-resistant strains.

We then sought to investigate the role of AbaR island in the multidrug resistance profile of strain AB5075. This AbaR contains 19 open reading frames (ORFs) with, besides transposon-related proteins, seven ORFs predicted to confer resistance to metals, three of unknown function, and the *sup* gene encoding a putative sulfate permease present in most AbaRs (Fig. 2A). Antibiotic susceptibilities were thus compared between the AB5075 parental strain and its AbaR-cured derivative, denoted AB5075-T (Table 1). As anticipated, the resistance profiles to 14 antibiotics belonging to five antibiotic classes were identical between the wild type and its AbaR-cured derivative. We then investigated the contribution to antibiotic resistance of a larger AbaR found only in another A. baumannii ST1 strain, the clinical strain AYE resistant to aminoglycosides, β-lactams (excluding imipenem, piperacillin-tazobactam, and ticarcillin-clavulanate), fluoroquinolones, and tetracycline and was of intermediate susceptibility to rifampicin (5, 22). This strain carries the largest AbaR described so far, AbaR1, at 86 kbp long, containing 25 genes putatively involved in resistance to antibiotics of several classes. By exploiting natural transformation of the strain AYE (15), we cured this strain of its AbaR1 island and compared the resistance profiles to 21 antibiotics of the parental strain to that of its AbaR-cured AYE-T derivative (Table 2). In contrast to strain AB5075, curing the AbaR island from the strain AYE resulted in restored susceptibility to aminoglycosides, tetracycline, and sulfonamide. The resistance armamentarium conferred by the 86-kbp-long AbaR1 may be attributed to specific genes that are listed in Table 2 according to previous analyses (4, 5, 23). Restored resistances to sulfonamides and tetracycline are consistent with the previous report of AbaR27 deletion in strain A424 leading to the loss of two copies of *sul1* genes and one *tetA* gene (8).

**TABLE 1:** Antimicrobial resistance profiles of A. baumannii strain AB5075 deleted of AbaR11 (AB5075-T) in comparison to the wild-type strain

Antibiotic^*a*^	Inhibition zone diam (mm)^*b*^	
	AB5075 WT	AB5075-T
AMK	20	20
GEN	11	12
TOB	10	10
CIP	6	6
PIP	6	6
TZP	6	6
TIC	6	6
TIM	6	6
CAZ	6	6
FEP	6	6
IMP	6	6
ATM	6	6
TET	18	18
SUF	6	6

a AMK, amikacin; GEN, gentamicin; TOB, tobramycin; CIP, ciprofloxacin; PIP, piperacillin; TZP, piperacillin plus tazobactam; TIC, ticarcillin; TIM, ticarcillin plus clavulanic acid; CAZ, ceftazidime; FEP, cefepime; IMP, imipenem; ATM, aztreonam; TET, tetracycline; SUF, sulfonamide.

b WT, wild type; AB5075-T, strain AB5075 deleted of AbaR11.

**TABLE 2:** Antimicrobial resistance profiles of A. baumannii strain AYE deleted of AbaR1 in comparison to the wild-type strain and genes carried by AbaR1 potentially involved in resistance

Antibiotic^*a*^	Inhibition zone diam (mm)		MIC (μg/ml)		MIC/C^*d*^ (μg/ml)		Predicted AbaR1 resistance gene involved^*e*^
	AYE WT^*b*^	AYE-T^*c*^	AYE WT	AYE-T	AYE WT	AYE-T	
AMK	8	21	64	8	—^*f*^	—	*aadB*, *aacC1*, *aphA1b*, *aacA*
GEN	6	19	128	6	—	—	
TOB	6	18	24	1.5	—	—	
KAN	—	—	>256	2	—	—	
STR	—	—	128	0.64	—	—	Two *aadA1* copies, *strA*, *strB*
CIP	6	6	—	—	—	—	
PIP	6	6	—	—	—	—	
TZP	14	15	—	—	—	—	
TIC	6	19	—	—	—	—	*bla*_VEB-1_, *bla*_oxa-10_^*g*^
TIM	15	18	—	—	—	—	*bla*_oxa-10_^*g*^
CAZ	6	11	>256	12	>256	3	*bla*_VEB-1_
FEP	6	16	256	12	>256	16	*bla*_VEB-1_
CTX	—	—	>32	>32	>32	>32	
ATM	6	11	—	—	—	—	*bla*_VEB-1_
IMP	28	28	—	—	—	—	
MEM	20	22	—	—	—	—	
SUF	6	23	—	—	—	—	Five *sul1* copies
SXT	—	—	>32	0.25	—	—	Five *sul1* copies, *dhfrI*, *dhfrX*
TET	—	—	128	8	—	—	*tetA(A)*, *tetA(G)*
CHL	—	—	>256	>256	—	—	*cmlA1*, *cmlA5*, *cmlA9*, *catA1*
RIF	—	—	12	8	—	—	*arr-2*

a AMK, amikacin; GEN, gentamicin; TOB, tobramycin; STR, streptomycin; KAN, kanamycin; CIP, ciprofloxacin; PIP, piperacillin; TZP, piperacillin plus tazobactam; TIC, ticarcillin; TIM, ticarcillin plus clavulanic acid; CAZ, ceftazidime; CTX, cefotaxime; FEP, cefepime; IMP, imipenem; MEM, meropenem; ATM, aztreonam; SUF, sulfonamide; SXT, trimethoprim-sulfamethoxazole; TET, tetracycline; CHL, chloramphenicol; RIF, rifampicin.

b WT, wild type.

c AYE-T, strain AYE deleted of AbaR1.

d C, cloxacillin at 250 μg/ml.

e According to references 4 and 5.

f —, not done.

g According to reference 24, *oxa-10* gene expression is probably weak.

In the AYE-T strain, partially decreased resistance to ticarcillin, ceftazidime, cefepime, and aztreonam was also observed. Only a partial loss of resistance to β-lactams upon AbaR1 curing is likely explained by the additional resistance mechanisms the AYE strain possesses, such as the overexpression of the *bla*_ADC-11_ gene encoding a narrow-spectrum chromosomal cephalosporinase (22, 24). In AYE-T strain ceftazidime, Etest values decreased from 12 μg/ml to 3 μg/ml in medium supplemented with cloxacillin (250 μg/ml), the latter known to inhibit the cephalosporinase, whereas no significant differences were observed for cefepime, which is not a substrate for ADC-11 (25). Other chromosomal resistance determinants most likely contribute to the reduced susceptibility to cefotaxime, for instance, the predicted new class A β-lactamase on AYE chromosome (24). Despite the four putative chloramphenicol resistance genes on AbaR1, their deletion did not modify the high intrinsic resistance of A. baumannii to this antibiotic mediated by efflux (26). Deletion of AbaR1 resulted in a minor change of rifampicin susceptibility.

Contrarily to the previous report on AbaR27 deletion, we did not observe for both strains, AB5075 and AYE, an increased resistance to fluoroquinolone of the AbaR-cured strains (8).

Beyond the role of AbaR in GC1 resistance, this method should allow investigation of other genomic islands found in A. baumannii as long as strains are transformable. For instance, as strains of GC2 are seemingly transformable, scarless removal of the AbGRI1 resistance island found in this clone is anticipated to be feasible (27, 28).

### Curing of AbaR and repair of the *comM* gene result in increased transformation ability.

**FIG 3:**
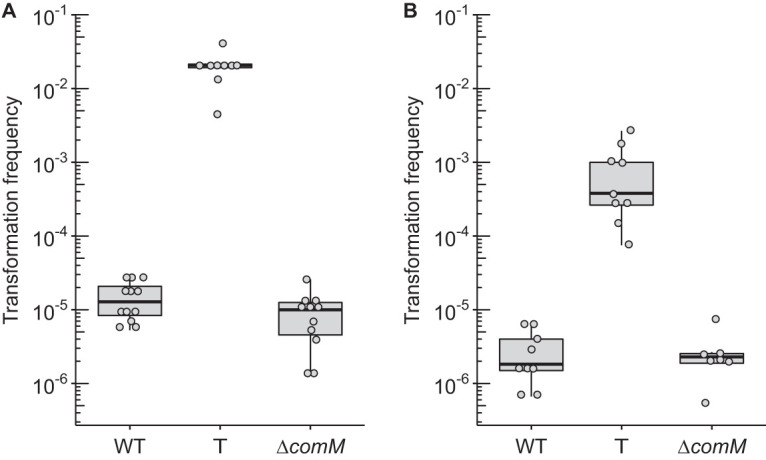
Transformation frequencies of A. baumannii strains AB5075 (A) and AYE (B) of a 3.8-kbp-long PCR product carrying a mutation in the *rpoB* gene conferring resistance to rifampicin (final concentration of 50 ng/μl, 125 ng per sample). For both strains, either the wild type or their “T” (ΔAbaR/repaired *comM*) or Δ*comM* derivatives were tested. At least seven independent transformation assays were performed on at least two separate occasions represented by separate dots and corresponding boxplots. All results were above the limit of detection (10^−8^). Pairwise comparisons using the nonparametric Mann-Whitney-Wilcoxon test (two-tailed) gave *P* values of at least <0.005 between transformation frequencies obtained for the wild-type/Δ*comM* strains and the T derivatives.

AbaR islands are inserted in the *comM* gene (4). In the nonpathogenic species A. baylyi, a mutation of the latter gene is associated with reduced transformability (9). We therefore pursued this by investigating the role of *comM* in A. baumannii as well as the physiological consequence of AbaR-island disruption of this gene. To do so, we performed transformation assays comparing the levels of transformation frequency between the wild-type and AbaR-cured strains using an *rpoB* mutation (I581F) conferring resistance to rifampicin as a genetic marker (Fig. 3). For both clinical strains, curing AbaR and thus restoring the *comM* gene increased significantly the ability to acquire the genetic marker, with a mean increase of approximately 1,000-fold in strain AB5075-T (Fig. 3A) and of approximately 300-fold in strain AYE-T (Fig. 3B). With one bacterium of 100 that performed natural transformation, strain AB5075-T presented the highest transformation ability (Fig. 3A). Using the same genome editing strategy as depicted in Fig. 1, we generated markerless deletions of the *comM* gene (Δ*comM*) in both A. baumannii clinical strains. Consistently, deletion of the *comM* gene in both strains resulted in transformation levels comparable to levels observed for the wild-type strains bearing a genuine insertion in the *comM* gene by the AbaR island (Fig. 3A and B). Thus, prior to acquisition of AbaR, both strains were transformable at higher levels, and the subsequent disruption generated transformation defects that are consistent with previous observations made in other species or genera (9, 10). The data show that the AbaR-inactivated *comM* gene, once cured of AbaR, is still functional. This suggests that it has not been affected by mutational drifts, confirming that acquisition of AbaR occurred relatively recently (4).

The fact that acquisition of the AbaR element in the *comM* gene alters the natural transformability of A. baumannii and its capacity for further acquisitions by natural transformation is intriguing. Interestingly, acquisition of AbaR and consequent *comM* interruption did not hinder GC1 subsequent acquisition of different capsule gene clusters or *gyrA* or *parC* alleles leading to fluoroquinolone resistance (29). The significance of lower transformability is not known, but theoretical work suggests that intermediate transformation rates allow bacteria to maximize fitness in fluctuating stressful environments such as repeated antibiotic stress (30).

### Conclusion.

By exploiting natural transformation, we set up a genetic system to easily edit the genome of A. baumannii. We used this strategy to perform small-gene deletion but also to cure large genomic islands in two multidrug-resistant clinical A. baumannii strains. Moving beyond predicted resistance, this system allowed experimental determination of the antibiotic resistance conferred by the islands. We conclude that acquisition of AbaR islands may increase A. baumannii strain resistance to antibiotics, but it comes at the cost of decreasing the cell’s ability to perform natural transformation by disrupting the *comM* gene. Thus, acquisition of AbaR may impact genome evolution of A. baumannii. From an applied perspective, the simultaneous deletion of AbaR and restoration of the *comM* function in two clinically relevant strains made them even more genetically amenable, blazing the trail for mutational analysis of these model A. baumannii strains.

## MATERIALS AND METHODS

### Bacterial strains, typing, and growth conditions.

The bacterial strains used in this study are listed in Table 3. Unless specified, A. baumannii isolates and strains were grown in Lennox broth (LB). All experiments were performed at 37°C. Antibiotic concentrations in selective media were 30 μg/ml for apramycin and 100 μg/ml for rifampicin.

**TABLE 3:** Acinetobacter baumannii strains used in this study

Strain name	Genotype	Description/phenotype	Reference
AB5075 WT	A. baumannii AB5075 wild type		31
AB5075 *comM*::*sacB_aacC4*	A. baumannii AB5075 *comM*::*sacB_aacC4*	Replacement of the AbaR island with the *sacB_aacC4* cassette, resistant to apramycin and sensitive to sucrose	This study
AB5075-T	A. baumannii AB5075 ΔAbaR11	Deletion of the AbaR11 island and a repaired *comM* gene, highly transformable strain	This study
AB5075 Δ*comM*	A. baumannii AB5075 Δ*comM*	Markerless 231-bp deletion of the *comM* gene	This study
AB5075 Rif^r^	A. baumannii AB5075 Rif^r^	Spontaneous *rpoB* mutant I581F; rifampicin resistant	This study
AYE WT	A. baumannii AYE wild type	Wild type	22
AYE *comM*::*sacB_aacC4*	A. baumannii AYE *comM*::*sacB_aacC4*	Strain with a replacement of the AbaR island with the *sacB_aacC4* cassette, resistant to apramycin and sensitive to sucrose	This study
AYE-T	A. baumannii AYE ΔAbaR1	Strain with a deletion of the AbaR1 island and a repaired *comM* gene, highly transformable and antibiotic susceptible strain	This study
AYE Δ*comM*	A. baumannii AYE Δ*comM*	Markerless 231-bp deletion of the *comM* gene	This study

### Construction of bacterial strains.

All oligonucleotides used in this study for genetic modification are listed in Table S1 in the supplemental material. Gene disruptions were performed using overlap extension PCR to synthesize large chimeric DNA fragments carrying the selection marker flanked by 2-kbp-long fragments that are homologous to sequences flanking the insertion site. The PCRs were performed with a high-fidelity DNA polymerase (PrimeStarMax; TaKaRa) (see “Detailed protocol of chimeric PCR for mutation in A. baumannii” in the supplemental material). When used as the template for the generation of PCR products, genomic DNA was extracted and purified using the Wizard Genomic DNA purification kit (Promega) according to the manufacturer’s guidelines. The AB5075 wild-type strain was naturally transformed with an assembly PCR product to generate the AB5075 *comM*::*sacB_aacC4* strain. The AYE *comM*::s*acB_aacC4* was then obtained by transforming the AYE wild-type strain with genomic DNA extracted from the AB5075 *comM*::*sacB_aacC4* strain. For both strains, transformants were selected on LB plates supplemented with apramycin, and susceptibility to sucrose was verified on M63 plates supplemented with 10% sucrose. The AB5075 and AYE *comM*::*sacB_aacC4* strains were naturally transformed with an assembly PCR product (obtained from assembly PCR on wild-type genomic DNA) to generate their T and Δ*comM* derivatives. Transformants were selected on M63 plates supplemented with 10% sucrose, and susceptibility to apramycin was verified on LB plates supplemented with apramycin. All chromosomal modifications were verified in transformants using colony PCR (cf. Table S1). Removal of AbaR and restoration of the *comM* gene were verified by Sanger sequencing.

The AB5075 rifampicin-resistant (Rif^r^) strain was generated by selecting spontaneous mutants after plating an overnight culture of the AB5075 wild-type strain on plates supplemented with rifampicin.

### Antibiotic susceptibility testing.

Susceptibility of the strains to a panel of 15 antibiotics (ticarcillin, ticarcillin-clavulanic acid, piperacillin, piperacillin-tazobactam, ceftazidime, cefepime, meropenem, imipenem, gentamicin, tobramycin, amikacin, ciprofloxacin, tetracycline, aztreonam, and sulfonamide) was evaluated by disc diffusion on Muller-Hinton agar (Bio-Rad, France) according the CA-SFM 2013 recommendations (https://resapath.anses.fr/resapath_uploadfiles/files/Documents/2013_CASFM.pdf). Inhibition values were interpreted according to CA-SFM 2013 breakpoints for all antibiotics but aztreonam, for which *Pseudomonas* spp. breakpoints are given. Susceptibilities of wild-type AYE and deleted AbaR1 mutant were also compared by Etest strips (bioMérieux, France) for tetracycline, streptomycin, trimethoprim-sulfamethoxazole, chloramphenicol, rifampicin, and β-lactams, including cefotaxime, ceftazidime, and cefepime. The strain Pseudomonas aeruginosa CIP 7110 was used as control for all antibiotic susceptibility testing.

### Transformation assay.

The method used in this study was described in reference 15 with the following modifications. After overnight incubation at 37°C on Lennox broth agar, the strains were cultured for a few hours in 2 ml of Lennox broth (LB) until an optical density at 600 nm (OD_600_) of at least 1 was reached. The bacterial broths were then diluted to an OD_600_ of 0.01 in phosphate-buffered saline (PBS). Then, small aliquots of bacterial suspensions (5 to 10 μl) were mixed with DNA substrate. To measure transformation frequency, a final PCR concentration of 50 ng/μl was used. The mixtures (2.5 μl) were deposited on the surface of 1 ml of transformation medium (2% agarose D3 [Euromexdex], 5 g/liter of tryptone, 2.5 g/liter NaCl), poured in 2-ml microtubes, and incubated overnight at 37°C. The next day, bacteria were recovered by resuspension in 300 μl of PBS. For genome edition experiments, the suspensions were spread on selective media (see “Construction of bacterial strains” for details). To measure transformation frequency, the bacterial suspensions were serial diluted and spread on LB agar plates without antibiotic or supplemented with rifampicin. All the transformation assays were performed on at least two separate experiments. On each occasion, at least three independent transformation reactions were conducted (three different transformation tubes). All the independent data points are plotted. As normality of the distribution of transformation frequency does not apply for transformation frequency analysis, nonparametric tests were performed (Mann-Whitney-Wilcoxon).

## Supplementary Material

Supplemental file 1
