# Supplementary material for: Scarless Removal of Large Resistance Island AbaR Results in Antibiotic Susceptibility and Increased Natural Transformability in Acinetobacter baumannii
Source: Antimicrob Agents Chemother. 2020 Sep 21;64(10):e00951-20. doi: 10.1128/AAC.00951-20 (PMC7508600; doi:10.1128/AAC.00951-20)
Supplement: Supplemental file 1 [file AAC.00951-20-s0001.pdf]

Title: Scarless removal of large resistance island AbaR results in antibiotic susceptibility and increased natural transformability in *Acinetobacter baumannii.*

Authors: Anne-Sophie Godeux[a,b], Elin Svedholm[a], Agnese Lupo[c], Marisa Haenni[c], Samuel Venner[d], Maria-Halima Laaberki[a,b,#,*], Xavier Charpentier[a,#].

a. CIRI, Centre International de Recherche en Infectiologie, Univ Lyon, Inserm, U1111, Université Claude Bernard Lyon 1, CNRS, UMR5308, ENS de Lyon , 69100, Villeurbanne, France

b. Université de Lyon, VetAgro Sup, 69280 Marcy l'Etoile, France.

c. Unité Antibiorésistance et Virulence Bactériennes, Université de Lyon - ANSES Site de Lyon, Lyon, France

d. Laboratoire de Biométrie et Biologie Evolutive, CNRS UMR5558, Université Claude Bernard Lyon 1, Villeurbanne, France

# Contributed equally

* Correspondence to Maria-Halima Laaberki, maria-halima.laaberki@vetagro-sup.fr

SUPPLEMENTAL MATERIAL

Table S1: primers used in this study

Detailed protocol of chimeric PCR for mutation in *A. baumannii*

SUPPLEMENTARY MATERIALS

Table S1: primers used in this study

Genetic construct	Name*	Sequence 5’ to 3’	Template; Annealing site
Chromosomal modifications using overlap extension PCR			
comM::sacB_aacC4	mlo-2 (P1)	GCCTGGAATTGTCATCAACAGAACC	AB5075 and AYE genomic DNA; *amt* gene = 2kb upstream of the *comM* gene
	mlo-3 (P2)	**GGCCCAATTCGCCCTATAGTGAGTCG**ATTGCTGATGCTGTATGGTGGGG	AB5075 and AYE genomic DNA; 5’ *comM* gene and adapter sequence reverse-complement to primer mazF-K7-F (bold and orange)
	mlo-4 (P3)	**GGGTTTGCTCGGGTCGGTGGCATATG**CAATGAATCCTTGTCCATGTGGG	AB5075 and AYE genomic DNA; 3’ *comM* gene and adapter sequence reverse-complement to primer mlo-1 (bold and blue)
	mlo-5 (P4)	GCATGTCTTGCTGTGCGTGTTGC	AB5075 and AYE genomic DNA; *ABUW_3681* gene = 2kb downstream of the *comM* gene
	mazF-K7-F (P5)	**CGACTCACTATAGGGCGAATTGGGCC**GCTTTCCAGTCGGGAAACCTG	pMHL-2 (Godeux et al., 2018) ; upstream *sacB* gene and adapter sequence (bold)
	mlo-1 (P6)	**CATATGCCACCGACCCGAGCAAACCC**CGCCAGGGTTTTCCCAGTCACGAC	pMHL-2 (Godeux et al., 2018) ; dowstream *aacC4* gene and adapter sequence (bold)
∆AbaR	mlo-6 (P7)	ACCGCCCCCAACCAGTGCAATTG	AB5075 and AYE genomic DNA *; comM* at AbaR insertion locus (annealing with mlo-7 is underlined)
	mlo-7 (P8)	CAATTGCACTGGTTGGGGGCGGTTCACACCCCAAACCGGGTGAAATTAC	AB5075 and AYE genomic DNA *; comM* at AbaR insertion locus (annealing with mlo-6 is underlined)
∆*comM*	asg-75 (P7’)	AATTTCTAGTGCACGCCG	AB5075 and AYE genomic DNA; 5’ *comM* gene (annealing with asg-76 is underlined)

	asg-76 (P8’)	CGGCGTGCACTAGAAATTCCAAACCGGGTGAAATTAC	AB5075 and AYE genomic DNA; 3’ *comM* gene (annealing with asg-75 is underlined)
Primers for control using colony PCR and for sequencing of the *comM* locus			
	mlo-80	CGGATCTTCGATGCTGGC	AbaR1 from AYE strain
	mlo-84	GCAACGATGTTACGCAGC	AbaR1 from AYE strain
	comM-For	CCACAATGGAACAAGAAGATGTCT	5’ of the *comM* gene
	comM-Rev	TTAAGAGTGATTACCTCGATAAGA	3’ of the *comM* gene
	mlo-29	TCATGAGCTCAGCCAATCGACTGG	End of ApraR cassette
	asg-61	CCACAGAATGATGTCACG	5’ of the *aacC4* gene
PCR product carrying the *rpoB* mutation as substrate for natural transformation			
	mlo-104	AAGATATCGGTCTCCAAGC	AB5075 genomic DNA; *rpoB* gene
	mlo-105	AGTACGGCCTTCAACGTCAT	AB5075 genomic DNA; *rpoB* gene

*as referred in Fig.1

Detailed protocol of chimeric PCR for mutation in *A. baumannii*

Two rounds of PCR are required to produce chimeric PCR products: a first PCR to add overlapping extensions to the DNA fragments and a second to assemble the first DNA fragments.

1. First PCR (Overlapping extension PCR)

PCR mix:

PCR mix	25µL reaction
DNA template [20ng to 100ng/µL]	1µL
Primer F 10µM	0.5µL
Primer R 10µM	0.5µL
PrimeSTAR premix (Takara)	12.5µL
H2O qsp 25µL	10.5µL

PCR cycling conditions :

Initial denaturation 98°C- 2 min.

Denaturation 98°C - 20 sec.
Annealing **55**°C – 20 sec.
Extension 72°C – 40 min.
(30 cycles)

Final extension 72°C – 5 min.

Then proceed to PCR purification after migration of the whole PCR reactions in buffered agarose gel stained with SYBR Safe (Thermofisher). The bands at the correct size are excised from the gel above a blue-light transilluminator (this is critical) and DNA extracted following instructions of the manufacturer (Omega Bio-Tek/VWR).

Alternatively, the PCR products are purified using magnetic beads following manufacturer's protocol (Ampure XP /Beckman Coulter) after a direct DpnI digestion of the genomic DNA in the PCR mix.

The purified PCR products are then analyzed (5µL) on agarose gel stained with ethidium bromide. Concentration should be at least 30ng/µL for each PCR products and a single band obtained.

2. Second PCR (assembly PCR)

This second round of PCR assembles the three PCR products with a ratio of 1/2/1 for PCR[P1 + P2] / PCR[P5 + P6] / PCR[P3 + P4] in the PCR mix using the P1 (forward primer) and P4 (reverse primer).

PCR mix:[DNA concentration for example]

PCR Assembly	50µL reaction
PCR[P1 + P2] [100ng/µL]	1
PCR[P5 + P6] [30ng/µL]	6
PCR[P3 + P4] [100ng/µL]	1
Primer P1 10µM	1
Primer P4 10µM	1
PrimeStar premix (Takara)	25
H_2O	15

Note that the total amount of PCR products is close to 500ng for a 50µL reaction, this can be up to 800ng-1µg, without exceeding a total of 10µL of first PCR products for a 50µL PCR reaction.

PCR cycling conditions :

Initial denaturation 98°C- 2’

Denaturation 98°C -20”
Annealing 55°C – 20” — 30 cycles
Extension 72°C – 1’ 30”

Final extension 72°C – 5’

Then proceed to PCR purification either after migration in buffered agarose gel stained using SYBR Safe (Thermofisher) followed by gel extraction (Omega Bio-Tek/VWR) or alternatively using Ampure XP beads (Beckman Coulter).
